# The gains of a 4‐week cognitive training are not modulated by novelty

**DOI:** 10.1002/hbm.24965

**Published:** 2020-03-17

**Authors:** Davina Biel, Tineke K. Steiger, Torben Volkmann, Nicole Jochems, Nico Bunzeck

**Affiliations:** ^1^ Institute of Psychology I University of Lübeck Lübeck Germany; ^2^ Institute for Multimedia and Interactive Systems University of Lübeck Lübeck Germany

**Keywords:** aging, cognitive training, memory enhancement, novelty, plasticity, VBM, VBQ

## Abstract

Cognitive training should not only improve performance of the trained task, but also untrained abilities. Exposure to novelty can improve subsequent memory performance, suggesting that novelty exposure might be a critical factor to promote the effects of cognitive training. Therefore, we combined a 4‐week working memory training with novelty exposure. Neuropsychological tests and MRI data were acquired before and after training to analyze behavior and changes in gray matter volume, myelination, and iron levels. In total, 83 healthy older humans participated in one of three groups: Two groups completed a 4‐week computerized cognitive training of a two‐back working memory task, either in combination with novel or with familiarized nature movies. A third group did not receive any training. As expected, both training groups showed improvements in task specific working memory performance and reaction times. However, there were no transfer or novelty effects on fluid intelligence, verbal memory, digit‐span, and executive functions. At the neural level, no significant micro‐ or macrostructural changes emerged in either group. Our findings suggest that working memory training in healthy older adults is associated with task‐specific improvements, but these gains do not transfer to other cognitive domains, and it does not lead to structural brain changes.

## 1 INTRODUCTION

In healthy older humans, cognitive abilities can be improved by cognitive training. While specific training gains appear to underline the brain's plasticity throughout the life‐span (Heinzel et al., 2014), some cognitive trainings go even further by demonstrating a transfer to untrained cognitive abilities (so called transfer effects). For instance, working memory trainings not only improved performance of the trained task (i.e., training gains), but they also improved fluid intelligence (Jaeggi, Buschkuehl, Jonides, & Perrig, 2008), verbal memory (Richmond, Morrison, Chein, & Olson, 2011), executive functions (Heinzel et al., 2016), and processing speed (Heinzel et al., 2016) (i.e., transfer effects). Along the same lines, effects of trainings that are based on video games have been shown to transfer to executive functions (Nouchi et al., 2012), processing speed (Nouchi et al., 2012), and working memory (Anguera et al., 2013). However, evidence in favor of transfer effects is equivocal and, therefore, the underlying processes remain unclear.

At the neural level, cognitive training has been associated with functional and anatomical effects. For instance, trainings can lead to increased dopamine release (Bäckman et al., 2017), increased striatal BOLD activity (Dahlin, Neely, Larsson, Backman, & Nyberg, 2008), and reduced hemodynamic activity in frontal brain regions (Heinzel et al., 2016). Interestingly, working memory training gains in older adults were most pronounced in those participants showing a pretest neural activity pattern that was similar to the one observed in younger controls (Heinzel et al., 2014), suggesting that interindividual variability may play an important role (see also Buitenweg, Murre, & Ridderinkhof, 2012). Moreover, a multi‐task video game led to functional changes in midline frontal theta (4–7 Hz) power and long‐range theta coherence as measured with EEG (Anguera et al., 2013). With regard to anatomical changes, increases in cortical thickness of frontal brain regions (i.e., left orbitofrontal cortex, right lateral orbitofrontal cortex, fusiform cortex; Engvig et al., 2010), and a preserved fractional anisotropy (FA; an indicator for the degree of restrictiveness of water molecules) of frontal white matter (Engvig et al., 2012) were reported. Similarly, the amount of working memory training correlated with FA increases in regions adjacent to the intraparietal sulcus and anterior part of the body of the corpus callosum (Takeuchi et al., 2010) further suggesting a role of interindividual differences in training gains and transfer effects.

Despite the above‐mentioned reports, several studies could not show a transfer of training gains to other domains (Owen et al., 2010; Redick et al., 2013; Shipstead, Redick, & Engle, 2010). While this may have several reasons, it appears obvious that the exact mechanisms and conditions under which transfer effects occur remain unclear. However, since striatal activity (Dahlin et al., 2008) and dopamine release within striatal areas (Bäckman et al., 2017) increase after working memory training, a positive effect to other domains that depend on striatal integrity seems feasible. In fact, the striatum is not only vital for working memory, but also associative memory (Bauer, Toepper, Gebhardt, Gallhofer, & Sammer, 2015), learning (Foerde & Shohamy, 2011), verbal memory (Steiger, Weiskopf, & Bunzeck, 2016), executive function (Leh, Petrides, & Strafella, 2010), and fluid intelligence (Rhein et al., 2014). Therefore, working memory training could have a positive effect on the dopaminergic circuit and therefore, enhance performance in the aforementioned cognitive domains.

Here, on the basis of a possible link between dopaminergic neuromodulation and training effects (Bäckman et al., 2017; Dahlin et al., 2008), we investigated whether novelty, which is also associated with dopamine release and synaptic plasticity, promotes training gains and transfer effects (Buitenweg et al., 2012). Indeed, novel information is supposed to activate a loop between the medial temporal lobe (MTL) and dopamine neurons of the substantia nigra/ventral tegmental area (SN/VTA; Lisman & Grace, 2005; Lisman, Grace, & Duzel, 2011). Specifically, a novelty signal is generated within the MTL, which is transmitted to SN/VTA neurons via a polysynaptic path. The SN/VTA, in turn, back projects to the MTL, where dopamine drives synaptic plasticity, learning, and memory processes. Evidence for such a loop has been provided by several studies in animals (reviewed in Lisman & Grace, 2005; Lisman et al., 2011) and, more recently, also humans (Bunzeck & Düzel, 2006; Bunzeck, Guitart‐Masip, Dolan, & Duzel, 2014; Wittmann, Bunzeck, Dolan, & Düzel, 2007). At the behavioral level, the presentation of novel images and the exposure to a novel virtual reality (VR) before a word‐learning phase improve subsequent memory performance (Fenker et al., 2008; Schomaker, van Bronkhorst, & Meeter, 2014) in humans. In animals, novelty exposure before and after the initial learning phase (Wang, Redondo, & Morris, 2010) drives memory performance via dopaminergic processes (Ballarini, Moncada, Martinez, Alen, & Viola, 2009; Li, Cullen, Anwyl, & Rowan, 2003; Moncada & Viola, 2007; Wang et al., 2010).

Therefore, training gains and transfer effects might be promoted by novelty and lead to structural brain changes within the dopaminergic mesolimbic system. In order to test this hypothesis, we combined a 4‐week two‐back working memory training with the presentation of novel nature movies (novelty group, NOV) or with the presentation of familiarized nature movies (familiarity group, FAM); a third passive control group did not receive any training task (CON). Before and after training, participants were assessed with a battery of neuropsychological tests (tapping into fluid and crystallized intelligence, attention and processing speed, verbal and numeric memory, and executive functions) and a computerized train ticket machine (testing for a transfer to rather unrelated everyday abilities). Further, we used magnetic resonance imaging (MRI) based micro‐ and macrostructural measures of gray matter (GM), myelination, and iron levels (Draganski et al., 2011; Weiskopf & Helms, 2008) before and after the training to further our understanding of the underlying neural processes. We had four major hypotheses: (a) Training improves performance of the trained task (i.e., training gains: Higher hit rates and faster reaction times over time); (b) on the basis of previous studies with a very similar task (Heinzel et al., 2016), we expected significant transfer effects in tests for fluid intelligence, verbal and numeric memory, processing speed and executive function; (c) we expected changes in microstructural and macrostructural integrity within mesolimbic brain regions; and (d) we expected these effects (a–c) to be enhanced by novelty. Additionally, we explored possible reasons for interindividual differences, that is, whether training gains and transfer effects relate to structural brain integrity at baseline and whether training gains further relate to personality traits (Big‐Five) or baseline cognitive abilities (MoCA).

## 2 METHODS

### 2.1 Participants

**Figure 1:**
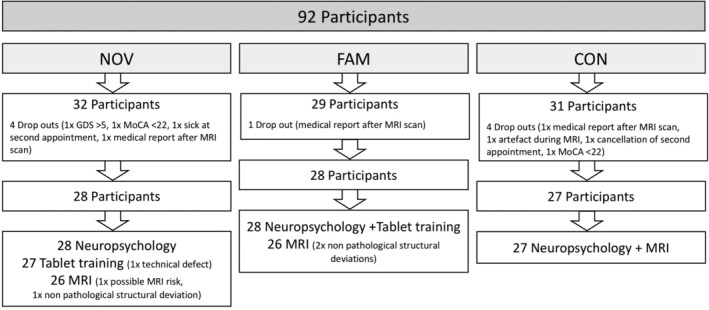
Flow chart of participant recruitment. Numbers indicate recruited participants, drop‐outs and the final sample. NOV, novelty group; FAM, familiarity group; CON, control group

In total, 92 healthy, right‐handed, German speaking older adults were recruited. However, nine participants were excluded due to a history of neurological, psychological or other severe physical disorders, drug abuse, CNS affecting medication intake (less than 2 weeks before testing), nonremovable metal implants or claustrophobia (for further details see Figure 1). Moreover, participants were excluded with >5 points in the Geriatric Depression Scale (GDS, maximum 15 points, >5 points indicates mild depression; Sheikh et al., 1991) and <22 points in the Montreal Cognitive Assessment (MoCA, maximum 30 points; Nasreddine et al., 2005; Freitas, Simões, Alves, & Santana, 2013). A value of 22 was chosen based on a study by Freitas et al. (2013), suggesting that it might be an appropriate cut‐off for mild cognitive impairment (MCI). Finally, 83 older adults (mean age 63.93 ± 8.54, 39 females) were included in the sample and randomly assigned into two experimental (novelty—NOV, familiarity—FAM) and one passive control group (CON). The NOV group included 28 participants (mean age 64.29 ± 9.69 years, 13 women, mean MoCA = 26.9 ± 1.91, 11.7 ± 1.54 mean years of school), the FAM group 28 participants (mean age 64.18 ± 8.10 years, 14 women, mean MoCA = 26.3 ± 2.16, 11.1 ± 1.88 mean years of school), and the CON group 27 participants (mean age 63.30 ± 7.99 years, 12 women, mean MoCA = 26.7 ± 2.18, 11.6 ± 1.6 mean years of school).

In total, 79 participants completed both MRI sessions and could, therefore, be included in further structural analyses. Finally, due to technical issues with one tablet, only the data of 55 instead of 56 training sessions were included in the analysis of the training task (Figure 1).

All participants were recruited through local newspaper announcements or the database of the Institute of Psychology (Greiner, 2015). All participants signed a written informed consent and received monetary compensation: Participants of the experimental groups (NOV and FAM) received 150 €, while participants of the CON group received only 60 €, since they were not participating in the 4‐week training period. The study was approved by the local ethical committee of the University of Lübeck, Germany, and in accordance with the Declaration of Helsinki. This study was not a registered trial.

### 2.2 Experimental design and procedure

**Figure 2:**
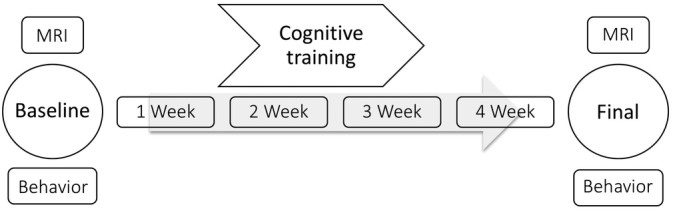
Experimental timeline. All participants completed a neuropsychological assessment and MRI measurements at baseline and 4 weeks after. Both experimental groups performed a 4‐week cognitive training. The passive control group only attended at baseline and 4 weeks later for posttest measurements without participating in a training program

First, all participants received a baseline examination at the University of Lübeck. This included a detailed neuropsychological assessment (duration ~2 hr, see below) and a structural MRI scan (duration ~1 hr, see below). Subsequently, both experimental groups (NOV and FAM) were instructed on how to perform the working memory task (see below) and how to use the tablet computer, which was provided for the following four training weeks. The CON group did not train the working memory task and, therefore, did not receive further explanations in this regard. Finally, after 4 weeks, all participants returned and completed a second neuropsychological assessment and structural MRI (Figure 2).

Note that participants of the experimental groups were not informed about the expected outcomes regarding different training manipulations (novel vs. familiar movies). Participants of the control group were informed that they were part of a control group; but, similar to the experimental groups, they were not informed about expected outcomes.

### 2.3 Cognitive assessment

Neuropsychological tests were acquired at two time points: Pretraining (t1) and posttraining (t2). They tapped into fluid and crystallized intelligence, verbal and numeric memory, processing speed, and executive functions. Fluid intelligence (Gf) was measured by the German Leistungsprüfsystem (LPS 50+) short version (for people aged 50–90 years; Sturm, Willmes, & Horn, 2015), which includes a battery of time restricted paper pencil tasks (duration ~30 min). For crystallized intelligence (Gc), the Mehrfachwahl‐Wortschatz‐Test (MWT) (Lehrl, 1995; Lehrl, Merz, Burkard, & Fischer, 1991) was applied; it provides 37 rows, each containing four pseudo‐words and one correct word, which has to be identified (with no time restriction).

Verbal memory was examined using the verbal learning and memory test (VLMT) (Helmstaedter, Lendt, & Lux, 2001). Here, a word list of 15 nonrelated items was verbally presented for five subsequent times. Each time, participants were asked to recall as many words as possible. Recalled words were noted from the examiner (total sum of correctly recalled words of all five runs refers to *VLMT learning* in further analysis). In a sixth run, an interference list of 15 words was verbally presented, which had to be immediately recalled. Subsequently, participants were asked to recall words from the initial list (without further verbal presentation from the examiner). After 20 min (again without further verbal presentation), the initial word list had to be recalled (*VLMT recall*). Consolidation loss (*VLMT cons*) is calculated by subtracting the amount of words remembered in the fifth round from *VLMT recall*. Finally, a recognition task was conducted. Here, words of the initial list were intermixed with words of the interference list and new words. The list was read out aloud and participants had to judge whether they recognized a word from the initial word list or not (*VLMT recognition*).

Numeric short‐term memory (working memory) was assessed by using a digit span forward and backward test (Wechsler, 1987). Participants had to remember verbally presented digits in the same or reversed order. Correct recall increased the digit span by one number. Forward started with three digits and ended with eight digits or after two errors within the same difficulty level; backward started with two digits and ended with seven digits or after two errors within the same difficulty level.

Processing speed and attention was tested using the standardized d2‐R test (Brickenkamp, Schmidt‐Atzert, & Liepmann, 2010). Here, participants had to mark as many targets (d's with exactly two dashes placed above or under the d) as possible within 14 rows containing d and p letters. After 20 s, participants had to switch to the next row. Following the test manual, the first and last row were not included into the analysis. After 4.6 min, the task was completed. We analyzed BZO (i.e., working speed), which refers to the number of marked items, and KL (i.e., concentration), which represents the corrected BZO score (BZO – [false positive + omissions] = KL) and is therefore a more sensitive marker for processing speed.

Executive functioning was tested using the trail making task (TMT; Reitan, 1992). First, participants had to connect randomly distributed circles containing numbers as fast as possible into the right order (Trail making task simple (TMT‐A), sustained attention; e.g., 1–2–3‐4). Subsequently, circles containing numbers and letters had to be connected in alternating order (trail making task complex (TMT‐B), divided attention; e.g., 1‐A‐2‐B‐3‐C).

To assess possible influences of personality traits, a short version of the Big‐Five inventory (BFI‐10) with five levels covering extraversion, agreeableness, conscientiousness, neuroticism, and openness to experience, was measured (Rammstedt, Kemper, Klein, Beierlein, & Kovaleva, 2013).

Finally, a task mimicking a ticket vending machine was adopted from a previous study (Sengpiel, 2016). Here, a task sheet containing four different ticket types with different difficulty levels (1× easy, 2× middle, 1× difficult) was handed out to the participants. For instance, the participant was instructed to select two group tickets for a specific fare zone (e.g., Berlin ABC) within the user interface of the ticket vending machine. There was no time restriction and only correctly selected tickets received one point. In total, four points could be achieved.

Parallel test versions were administered in a counterbalanced order for the LPS 50+, MWT, VLMT, and the ticket vending machine.

In total, 11 participants had to be excluded from the respective analysis. Six were excluded since their behavioral performance was more than three standard deviations (*SD*) above the group's mean at t1 or t2. More precisely, for VLMT recognition: One participant (CON group); for digit span forward: One participant (CON group); for TMT‐A: One participant (FAM group); and for TMT‐B: Three participants (one NOV group and two CON group). Moreover, one participant (NOV group) did not complete the VLMT recognition and, therefore, was also excluded. Finally, four participants had to be excluded for technical reasons from the analysis of the ticket vending machine data (one participant FAM and three participants CON group). Table 1 shows the number of subjects included in the analyses for each task and group.

**Table 1:** Number of subjects included in the analysis of each test

Analysis	Number of participants (total)	Number of participants (NOV)	Number of participants (FAM)	Number of participants (CON)
LPS 50+	83	28	28	27
MWT	83	28	28	27
VLMT learning	83	28	28	27
VLMT recall	83	28	28	27
VLMT consolidation	83	28	28	27
VLMT recognition	81	27	28	26
d2‐R BZO	83	28	28	27
d2‐R KL	83	28	28	27
Digit span forward	82	28	28	26
Digit span backward	83	28	28	27
TMT‐A	82	28	27	27
TMT‐B	80	27	28	25
Ticket vending machine	79	28	27	24

### 2.4 Cognitive training

The cognitive training comprised a total of 12 training sessions over 4 weeks. Participants were instructed to perform three training sessions each week, ideally with 1 day between each session. The tablets were programmed in a manner that it was not possible to perform more than one training a day or more than three trainings per week. If the participants tried to perform an additional training, a specific note was displayed. Moreover, the tablet contained a calendar app with highlighted days of already finished trainings, to help the participants to keep an overview of the training intervals. In most cases, participants were tested at t2 within the consecutive week of the last training session (see results).

**Figure 3:**
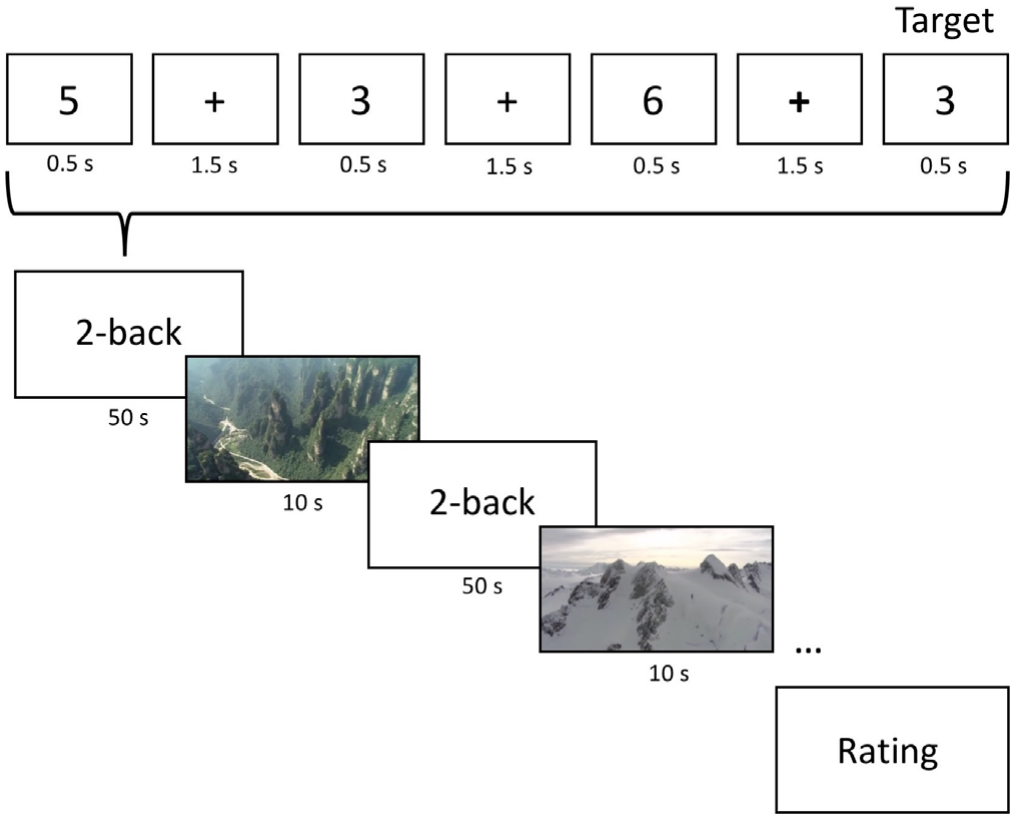
Cognitive training task. In the two‐back task, subjects had to identify those digits that were identical to the one shown two items before. The two‐back task was intermixed with video sequences. The NOV group watched novel movie sequences, while the FAM group watched five repeating movies during the whole training period

A typical two‐back working memory task was used in this study. Digits were subsequently displayed on the tablet (for 500 ms, followed by a fixation cross for 1,500 ms) and subjects had to identify those digits that were identical to the one shown two items before. Responses were given by button presses on the tablet. Each training session consisted of nine runs, 4 min each. In these 4 min, 50 s periods of the two‐back task were followed by 10 s long silent nature movies (no overt task required except watching, see below). After each run (i.e., four two‐backs plus movies), an interval scale appeared on the tablet prompting participants to rate the previously presented four movies (range from very uninteresting to very interesting). After the rating, the task could be continued by pressing a button (Figure 3). After approximately 36 min (short breaks after the rating scale excluded), the session was completed.

Both experimental groups (NOV and FAM) only differed in the presented movie types. While the NOV group watched, in total, 432 novel (i.e., unique) movies (36 per session), the FAM group watched the same five repeating movie clips over all 12 training sessions (also 432 times in total). In other words, the movies in the FAM group were initially novel but quickly became familiar within the first training session.

The movies depicted nature scenes from different continents (i.e., Africa, America, Asia, Europe, and Oceania), each further divided into different regions. For the NOV group, the 432 movie clips were well balanced and randomized across the different locations. No humans were shown during the sequences and emotional content was avoided (e.g., hunting predators). In both groups, participants were instructed to carefully watch the movies (no other task was required). To further ensure that participants payed attention to the movies (especially in the FAM group), they were only 10 s long and randomly presented.

### 2.5 Image acquisition

Structural MRI was performed at the University of Lübeck using a 3T Siemens Magnetom Skyra scanner equipped with a 64‐channel head coil. Whole‐brain multiparameter mapping (MPM; scanning time ~20 min) was acquired as reported previously (Weiskopf & Helms, 2013) using multi‐echo 3D fast low angle shot (FLASH) at 1 mm isotropic resolution. The volumes (voxel size 1 × 1 × 1 mm, matrix 176 × 256) were acquired for T1, proton density (PD) and magnetization transfer (MT) weightings. The weightings differed in TE, TR, and flip angles. T1‐weighted: Six echo times (TE = 2.2, 4.7, 7.2, 9.7, 12.2, and 15 ms), TR = 19 ms and flip angle = 20°; PD‐weighted: Eight echo times (TE = 2.2, 4.7, 7.2, 9.7, 12.2, 15, 17.5, and 20 ms), TR = 24 ms, flip angle = 6°; MT‐weighted: Six echo times (TE: 2.2, 4.7, 7.2, 9.7, 12.2, and 15 ms), TR = 37 ms, flip angle = 6°. A Gaussian MT‐pulse following Siemens product sequences was used. To shorten the scan duration, GRAPPA with an acceleration factor 2 and a partial Fourier acquisition 6/8 were applied. Subsequently, two runs of diffusion weighted imaging (DWI) using an EPI sequence were performed during the same scanning session (scanning time ~16 min). The images were later used for further analysis (results will be reported elsewhere).

MR data were further processed using the Statistical Parameter Mapping framework (SPM 12, Wellcome Trust Center for Neuroimaging, London) and MATLAB software (R2014b version, MathWorks). R2* maps were calculated through a regression of the log signal from the PD‐weighted echoes. Averaging the set of echoes for each weighting increased the signal‐to‐noise‐ratio for estimation of the MT map (Helms & Dechent, 2009). The semiquantitative MT map was calculated as described by Helms et al. (Helms, Dathe, & Dechent, 2008; Helms, Dathe, Kallenberg, & Dechent, 2008). Subsequently, images were slightly manually re‐orientated using SPM *Check Reg* and *Display* options (Ashburner, 2015).

### 2.6 Voxel‐based morphometry and voxel‐based quantification

GM volumes were processed and analyzed following a protocol for voxel‐based morphometry using SPM's batch system (VBM; Ashburner & Friston, 2000; Ashburner, 2015). Since MT maps provide increased contrast for subcortical regions (Helms, Draganski, Frackowiak, Ashburner, & Weiskopf, 2009; Lorio et al., 2014), in a first step, they were used for segmentation of the different tissue groups. Subsequently, images of GM, WM, and cerebrospinal fluid (CSF) were generated in native space (Ashburner & Friston, 2005). Applying high dimensional warping, images were then normalized to MNI space using the diffeomorphic registration algorithm (DARTEL) implemented in SPM, scaled by the Jacobian determinants of the deformation field and smoothed with an isotropic Gaussian Kernel of 6 mm full width at half maximum (FWHM). Finally, the resulting smoothed, modulated and normalized images were used for statistical analysis.

Voxel‐based quantification (VBQ) analysis provides sensitivity to tissue microstructure and is therefore well suited to test differences in R2* and MT, which are sensible marker for alterations in subcortical brain regions. VBQ was processed using the open source hMRI toolbox (Tabelow et al., 2019) embedded in the SPM framework. The toolbox combines both the VBQ (Draganski et al., 2011) and the MPM (Helms et al., 2009; Helms, Dathe, Kallenberg, & Dechent, 2008; Weiskopf et al., 2011, 2013) approach. Using the integrated processing pipeline of the toolbox, the previously generated MT maps were further processed using the modules *tissue segmentation* (GM, WM, and CSF), *DARTEL*, *creation of templates*, and *normalization* to MNI space. Subsequently, tissue‐weighted smoothing with a FWHM isotropic Gaussian kernel of 3 mm (Draganski et al., 2011) was performed. Resulting images of R2* and MT (each separately in GM and WM subspace) were used to indirectly test for differences in iron levels (R2*) and myelination (MT) of brain tissue (see also Callaghan et al., 2014; Draganski et al., 2011).

### 2.7 Statistical analysis

For both experimental groups, corrected hit rates (cHRs) of the two‐back training task were calculated (range −1 to +1, while 1 means perfect discrimination between targets and no targets). The cHRs of correctly identified two‐back trials were defined as follows:

$$\mathrm{cHR}=\frac{\text{Hits}}{\text{Possible correct hits}}-\frac{\text{False alarms}}{\text{Possible false alarms}}.$$

The reaction times (RTs) for hits within each training session were averaged for subsequent between‐subject analyses. RTs of 2 SD above and below the subject's mean were excluded. Further, participants with more than 3 SD above the overall mean of a specific neuropsychological test were excluded from the respective analysis.

To test for possible group differences at the beginning of the training, a *t*‐test for independent samples with the between‐subject factor *group* (NOV, FAM) was conducted on cHRs and RTs, respectively, from the first training session. The effects of the training and novelty on cHR and RT, respectively, were analyzed using a two‐way mixed‐design ANOVA (2 × 2) with the between‐subject factor *group* (NOV, FAM) and the within‐subject factor *time* (start, end).

Transfer effects to other cognitive domains and possible effects of novelty were investigated using separate two‐way mixed‐design ANOVAs (3 × 2) for repeated measurements with the between‐subject factor *group* (NOV, FAM, CON) and the within‐subject factor *time* (t1, t2).

The relationship of group and movie rating was calculated using a *t*‐test for independent samples with the between‐subject factor group (NOV vs. FAM). Further, correlation analyses were performed to investigate possible relationships between (a) training gains (cHR start/end differences) and MoCA scores, (b) training gains and personality traits (i.e., extraversion, agreeableness, conscientiousness, neuroticism, openness to experience), (c) transfer effects and MoCA scores, and (d) transfer effects and personality traits.

Finally, post hoc *t*‐tests were used when applicable and Bonferroni corrected with an adjusted alpha level of .016 (0.5/3—comparison between the three groups). Similarly, correlation analyses were adjusted for the number of comparisons (see results). All behavioral analyses were performed using IBM SPSS Version 24.

Possible structural brain changes between pretest and posttest were analyzed with SPM12 using MATLAB 2014b. Main effects of the factor *group* and *time* were calculated using a full factorial design. A flexible factorial design—well suited for models with repeated measurements—was conducted for analyses of interaction effects between *group* and *time*. The model comprised three factors (factor 1: *Subject*, factor 2: *Group* [three levels: NOV, FAM and CON], factor 3: *Time* [two levels: t1 and t2]). Contrasts were defined as described by Gläscher and Gitelman (2008). To further investigate possible training effects on the structural integrity of the basal ganglia, a mask containing the putamen, caudate, pallidum, and nucleus accumbens was applied. The mask was taken from the Harvard‐Oxford‐Atlas (50% probability mask), implemented in the FMRIB Software Library (FSL; Jenkinson, Beckmann, Behrens, Woolrich, & Smith, 2012).

A multiple regression analysis was conducted including all three groups in order to test for relationships between baseline structural integrity and possible transfer effects. For this, pre/post differences of each neuropsychological test were correlated with baseline MRI images (i.e., VBM and VBQ). For both experimental groups (NOV + FAM), a second multiple regression analysis was calculated. Here, baseline MRI and training gains (cHR start/end differences) were correlated.

Since there were no significant training effects for TMT‐A or TMT‐B and in order to reduce the number of comparisons, for the structural analyses, scores of digit span forward and backward and scores of TMT‐A and TMT‐B were combined (i.e., times were added). Due to two outliers (>3 SD) in the combined TMT, separate linear regression analyses of VBM and VBQ were conducted.

Data were normally distributed for most groups and time points, except for a few cases. For instance, normality assumption was violated at t2 for the digit span forward data in the FAM group. Moreover, homogeneity of variance was given, except for data from the ticket vending machine. Since there are no suitable nonparametrical tests for mixed design ANOVAs and given the rather large sample size, no additional analyses were conducted. However, regarding the movie ratings, the nonparametric Mann–Whitney *U* test was calculated. For the regression analyses, including the BFI‐10, Kendall's tau nonparametric correlation coefficient was used.

## 3 RESULTS

### 3.1 Behavioral data

In total, most participants completed all training sessions as instructed (NOV group mean: 11.79 ± .50 sessions; FAM group mean: 11.61 ± .88 sessions). More precisely, in the NOV group, 12 sessions were completed by 23 participants, 11 sessions were completed by 4 participants, and 10 sessions were completed by 1 participant. In the FAM group, 12 sessions were completed by 21 participants, 11 sessions were completed by 5 participants, 10 sessions were completed by 1 participant, and 8 sessions were completed by 1 participant. Two *t*‐tests for independent samples on cHRs and RTs, respectively, for the first training session did not reveal significant effects for cHRs or RTs (*cHR*: *t*[53] = .0456, *p* = .964, Cohen's *d* = .0123; *RTs*: *t*[53] = .766, *p* = .447, Cohen's *d* = .207) indicating no statistically significant differences between groups at baseline.

**Figure 4:**
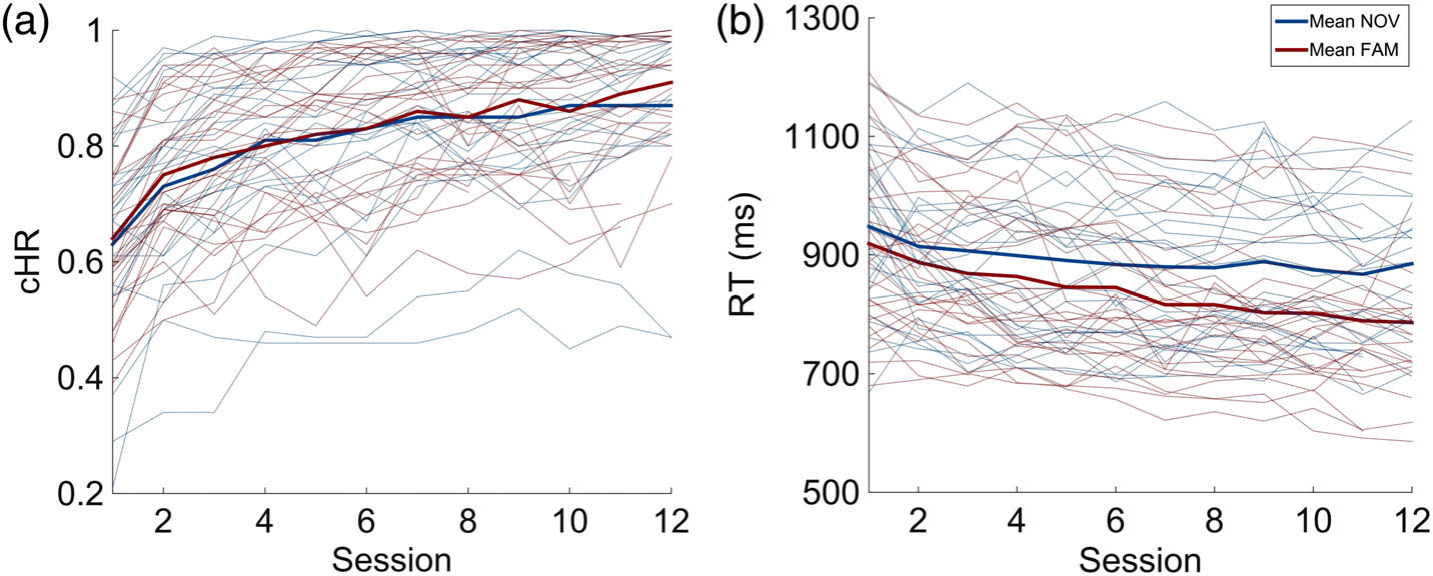
Training performance over time. Graphs show an increase of corrected hitrate (cHR; a) and a decrease of reaction time (RT; b) for both experimental groups over time. Thicker lines represent mean values of the respective group, thinner lines represent individual performances. Note, that not all participants completed the 12 training sessions (NOV: 22 = 12 sessions, 4 = 11 sessions, 1 = 10 sessions; FAM: 21 = 12 sessions, 5 = 11 sessions, 1 = 10 sessions, 1 = 8 sessions)

A 2 × 2 ANOVA on *cHR* with the between‐subject factor *group* (NOV, FAM) and the within‐subject factor *time* (start, end) revealed a main effect of time (*cHR*: *F*[1,53] = 227.293, *p* < .001, partial η^2^ = .811). Post hoc comparison revealed higher *cHR* at the end of the training as compared to the beginning (*cHR* start mean: .64; *cHR* end mean: .88; Figure 4a). However, there was no main effect of group (*F*[1,53] = .027, *p* = .871, partial η^2^ = .001) and no significant group by time interaction (*cHR*: *F*[1,53] = .052, *p* = .820, partial η^2^ = .001). A similar pattern emerged in the 2 × 2 ANOVA on *RT*: There was a main effect of time (*F*[1,53] = 51.830, *p* < .001, partial η^2^ = .494), which was driven by faster *RTs* for the end as compared to the beginning of the training (*RT* start mean: 933.05 ms; *RT* end mean: 831.43, Figure 4b). However, there was no main effect of group (*F*[1,53] = 1.983, *p* = .165, partial η^2^ = .036) and no group by time interaction (*RT*: *F*[1,53] = 1.736, *p* = .193, partial η^2^ = .032).

For the NOV group, the average number of days between t1 and t2 assessment was 31.5 ± 4.38, for the FAM group 31.2 ± 4.17, and for the CON group (which did not participate in a training program) 30.8 ± 4.78 days. The average number of days between the last session of the training and t2 was 3 ± 1.78 days for the NOV group and 3.11 ± 1.37 days for the FAM group. Due to technical issues, the last analysis includes only data from 37 participants (NOV *n* = 18, FAM *n* = 19).

Separate 3 × 2 ANOVAs with the factors group (NOV, FAM, CON) and time (t1, t2) revealed main effects of *time* for Gf *(LPS 50+)*, verbal memory *(VLMT learning*, *free recall and recognition)*, processing speed *(d2‐R working speed and concentration)*, and executive function *(TMT‐A and TMT‐B*; see Table 2 for statistical values). Post hoc *t*‐tests revealed higher scores for all these tests after training as compared to before training (all *p*'s < .008), indicating performance improvements over time (Table 2).

**Table 2:** Results and statistical values of the 2×3 ANOVAs

Domain	Test	*F* statistic	*p* value	Effect size partial η^2^
*Main effects time*				
Fluid intelligence	LPS 50+	*F*[1,80] = 76.18	**<.001**	0.488
Crystallized intelligence	MWT	*F*[1,80] = 0.791	.376	0.010
Verbal memory	VLMT learning	*F*[1,80] = 49.756	**<.001**	0.383
	VLMT free recall	*F*[1,80] = 21.323	**<.001**	0.210
	VLMT consolidation	*F*[1,80] = 1.476	.228	0.018
	VLMT recognition	*F*[1,78] = 19.203	**<.001**	0.198
Processing speed	d2‐R working speed (BZO)	*F*[1,80] = 48.259	**<.001**	0.376
	d2‐R concentration (KL)	*F*[1,80] = 53.775	**<.001**	0.402
Executive functioning	TMT‐A	*F*[1,79] = 6.976	**.01**	0.081
	TMT‐B	*F*[1,77] = 6.099	**.016**	0.073
Numeric memory	Digit span forward	*F*[1,79] = 0.692	.408	0.009
	Digit span backward	*F*[1,80] = 1.998	.161	0.024
Every‐day ability	Ticket vending machine	*F*[1,76] = 2.106	.151	0.027
*Main effects group*				
Fluid intelligence	LPS 50+	*F*[2,80] = 0.575	.565	0.014
Crystallized intelligence	MWT	*F*[2,80] = 0.509	.603	0.013
Verbal memory	VLMT learning	*F*[2,80] = 1.134	.327	0.028
	VLMT recall	*F*[2,80] = 1.122	.331	0.027
	VLMT consolidation	*F*[2,80] = 0.267	.767	0.007
	VLMT recognition	*F*[2,78] = 0.587	.558	0.015
Processing speed	d2‐R working speed (BZO)	*F*[2,80] = 1.303	.278	0.032
	d2‐R concentration (KL)	*F*[2,80] = 0.816	.446	0.020
Executive functioning	TMT‐A	*F*[2,79] = 0.169	.845	0.004
	TMT‐B	*F*[2,77] = 0.496	.611	0.013
Numeric memory	Digit span forward	*F*[2,79] = 1.531	.223	0.037
	Digit span backward	*F*[2,80] = 0.606	.548	0.015
Every‐day ability	Ticket vending machine	*F*[2,76] = 0.045	.956	0.001
Interactions (time*group)				
Fluid intelligence	LPS 50+	*F*[2,80] = 1.061	.351	0.026
Crystallized intelligence	MWT	*F*[2,80] = 0.097	.907	0.002
Verbal memory	VLMT learning	*F*[2,80] = 3.254	**.044**	0.075
	VLMT recall	*F*[2,80] = 2.880	.062	0.067
	VLMT consolidation	*F*[2,80] = 1.310	.276	0.032
	VLMT recognition	*F*[2,78] = 1.026	.363	0.026
Processing speed	d2‐R working speed (BZO)	*F*[2,80] = 3.588	**.032**	0.082
	d2‐R concentration (KL)	*F*[2,80] = 2.590	.081	0.061
Executive functioning	TMT‐A	*F*[2,79] = 0.844	.434	0.021
	TMT‐B	*F*[2,77] = 0.966	.385	0.024
Numeric memory	Digit span forward	*F*[2,79] = 1.345	.267	0.033
	Digit span backward	*F*[2,80] = 0.911	.406	0.022
Every‐day ability	Ticket vending machine	*F*[2,76] = 0.110	.896	0.003

*Note*: For each neuropsychological test, a 2 × 3 ANOVA with the factors time (t1, t2) and group (NOV, FAM, CON) was calculated. Significant *p* values are highlighted in bold letters.

No main effects of *time* were found for Gc (*MWT*), a subtest of the verbal memory task (*VLMT consolidation*), numeric memory (*digit span forward* and *backward*), and the *ticket vending machine* (Table 2).

There were no main effects of group for any score (Table 2).

Interactions (time*group) could be revealed for processing speed (*d2‐R working speed, BZO*: *F*[2,80] = 3.588, *p* = .032, partial η^2^ = .082), and verbal memory (*VLMT learning*: *F*[2,80] = 3.254, *p* = .044, partial η^2^ = .075). There were no other interactions (Table 2).

**Figure 5:**
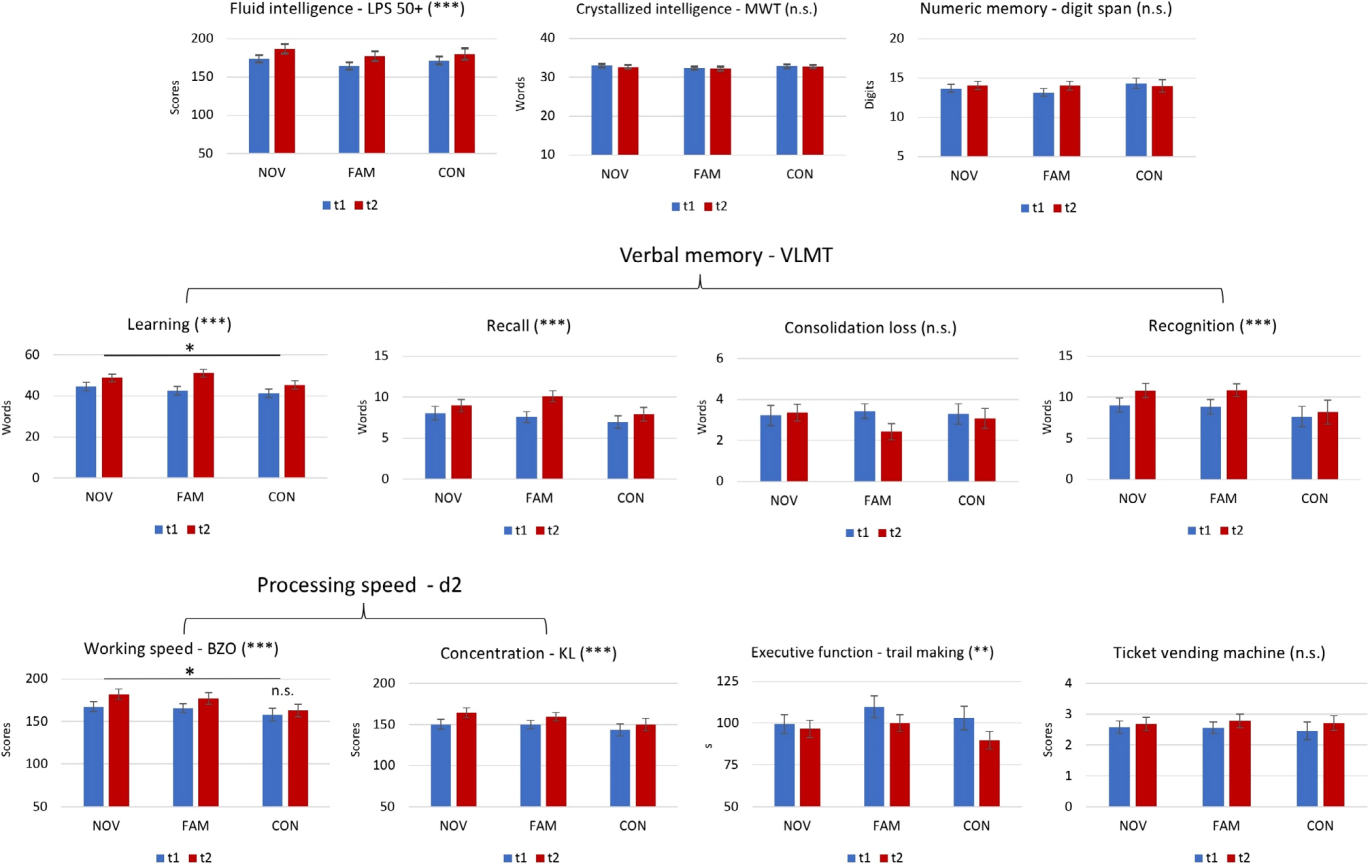
Results of the neuropsychological assessment. Significant main effects of time (t1 vs. t2) are marked in brackets (*p < .05; **p < .01; ***p < .001; n.s. = not significant). For displaying purposes, values of digit span forward and backward (= numeric memory) as well as TMT‐A and TMT‐B (= executive function) were combined. A significant interaction of group and time was only observed for working speed (BZO) and VLMT learning (see text), which are marked with an asterisk

Post hoc analysis (paired sample *t*‐tests) revealed a significant difference for the pre versus post comparison in *d2‐R working speed* (BZO) in the NOV and FAM group (NOV: *p* < .001, FAM: *p* < .001) but not the CON group (*p* = .074). For the verbal memory interaction (*VLMT learning*), a significant difference for the pre vs. post comparison could be revealed for all three groups (NOV: *p* = .008, FAM: *p* < .001, CON: *p* = .006); see Figure 5), but the comparison of the differences between groups did not survive Bonferroni correction (*p* > .016). That means, despite a significant time × group interaction (*p* = .04), post hoc tests could not pinpoint significant differences between groups for VLMT learning.

**Figure 6:**
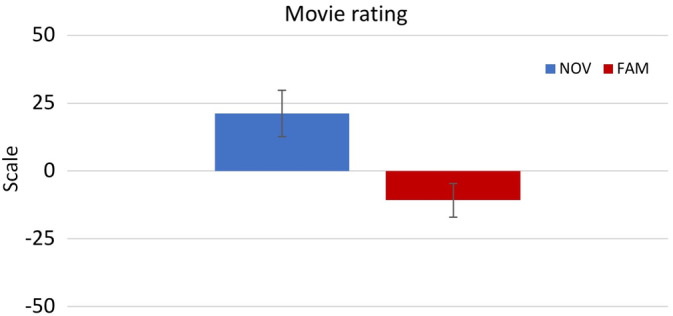
Main effect of movie rating. Participants of the NOV group rated movie sequences, which were presented during the training session, more positively (mean 21.22) than participants of the FAM group (mean −10.75)

A nonparametrical equivalent for a *t*‐test for independent samples (i.e., Mann–Whitney *U* test) on movie ratings revealed a main effect *group* (U = 196, *p* = .002, Cohen's *d* = .821). Post hoc analysis showed that novel movies were rated more positively as compared to familiar movies (Figure 6).

Finally, exploratory correlation analyses (first the NOV and FAM group separately, followed by across groups) of behavioral training gains (cHR start/end differences) and MoCA scores, did not reveal any significant effects. Similarly, there was no significant correlation between training gains and personality traits (all *p*'s > .05).

Similarly, correlation analyses for pretraining versus posttraining difference of the cognitive tests and MoCA scores, and pre vs. post training difference of the cognitive tests and personality traits also did not reveal any significant effects (all *p*'s ≥ .01, and therefore, did not survive Bonferroni corrections with adjusted alpha levels of .05/13 and .05/65, respectively).

### 3.2 Structural data

In a first full‐factorial design with the factors group (NOV, FAM) and time point (pretraining, posttraining), no main effects were found for group or time (FWE, *p* < .05 whole brain) for measures of GM volume (VBM), myelination (VBQ on MT maps) and iron levels (VBQ on R2* maps). Subsequently, a flexible factorial design did not reveal any group by time interactions (FWE, *p* < .05 whole brain). To further examine possible effects of the cognitive training on subcortical brain regions, a mask for the basal ganglia was applied for VBM and VBQ (R2* and MT maps) with a more liberate threshold (FWE, *p* < .05 cluster level; cluster size >50 voxel). Again, these analyses did also not reveal any significant effects.

Finally, exploratory multiple regression analyses (FWE, *p* < .05 whole brain) were conducted for baseline MRI data (VBM and VBQ) and pre/post differences of the cognitive assessments (including training gains). No statistically significant effects could be revealed.

## 4 DISCUSSION

We investigated the behavioral and neural effects of a 4‐week cognitive training and their potential modulation by novelty. As expected, the training improved performance in the two‐back working memory task but these effects did not transfer to other untrained domains. Although novel movies were rated as more positive than repeated ones, novelty did not drive performance in the trained or in any untrained task. At the neural level, no pre vs. post training differences could be observed in any microstructural and macrostructural modality (R2*, MT, and GM). Together, our findings suggest that, in healthy older adults, the benefits of a 4‐week working memory training do not transfer to other untrained abilities, and a combined passive exposure to novelty has no further promoting effects. In the following, we will discuss possible explanations of our findings and conclude that, in the light of our study, the effects of cognitive training appear rather weak.

Several studies have demonstrated beneficial effects of cognitive training to untrained abilities (Heinzel et al., 2016; Jaeggi et al., 2008; Salminen, Kuhn, Frensch, & Schubert, 2016). However, more recent work casts doubts about the success with regard to transfer effects (Bellander et al., 2017; Owen et al., 2010; Rabipour & Raz, 2012; Redick et al., 2013; Simons et al., 2016). Here, we can show that benefits of a 4‐week cognitive training (two‐back task) are evident in the trained task (Figure 4), which agrees with most training studies, but they do not transfer to other near (i.e., working memory) or far (i.e., verbal memory) cognitive domains. At least a near transfer effect would have been plausible given the notion that brain plasticity is restricted to the trained task (Lindenberger, Wenger, & Lövdén, 2017) and transfer to other domains is only possible if commonalities to the training task exist (Lövdén, Bäckman, Lindenberger, Schaefer, & Schmiedek, 2010). Therefore, it remains unclear under which conditions transfer effects (near and far) may occur.

In agreement with the notion of a transfer effect, Heinzel et al. (2016) could show that a similar version of a working memory training improves processing speed, fluid intelligence, and executive functions. While BZO (working speed in the d2‐R‐test) also improved by training in our study, it does not consider false positives and omissions. Therefore, KL appears to be a more suitable measure of concentration in the d2‐R‐test, which however, in our study did not benefit from training (Figure 5). Another critical difference to Heinzel et al. (2016) is that we did not tailor the training adaptively to the participants’ performance level. While individualized trainings appear to be a critical factor for training success and transfers effects (Buitenweg et al., 2012), we aimed to avoid differences between groups with regard to training demands. In other words, we expected novelty to drive training success (see below for an explanation), which, in case of individual adaptations, could have resulted, for instance, in faster digit presentations or longer delays (depending on how an adaptation is realized) between the novelty and familiarity group. This was avoided by a constant training paradigm for all participants. Future studies may take this aspect into account more rigorously, especially in order to avoid ceiling effects (Figure 4).

Another explanation for no transfer effects in our study may relate to task complexity. For instance, Jaeggi et al. (2008), who could demonstrate transfer effects in older adults on Gf, have used a working memory training task that required both visual and auditory modalities, and therefore, relied much more on binding processes and attentional control. Similarly, playing a video game for 1 month (three times per week) only led to transfer effects in a working memory task when it required multitasking but not in a simpler single version (Anguera et al., 2013). Interestingly, these training effects persisted for 6 months and were associated with functional changes in midline frontal theta (4–7 Hz) power and long‐range theta coherence as measured with EEG.

The absence of transfer effects may also relate to training duration and sample size. However, several previous studies—showing training gains and transfer effects—included fewer subjects and have the same number of sessions as our study (Anguera et al., 2013; Heinzel et al., 2014, 2016). Furthermore, expected transfer effects do not necessarily need to be small. Instead, in (Heinzel et al., 2016) transfer effects after a 4‐week cognitive training were rather strong (partial η^2^ > 0.14, as defined in Cohen, 1988). Specifically, the working memory training in their study had transfer effects on performance in a test on processing speed (d2) with a partial η^2^ of 0.28, a test on executive functions (Stroop test) with a partial η^2^ of 0.19, and a test on fluid intelligence (LPS) with a partial η^2^ of 0.14. Moreover, based on their findings, a power analysis (G*Power) shows that our study involved a sufficient number of subjects in order to replicate their findings with a power of at least 80% (i.e., processing speed (d2): 13 per group, executive functions (Stroop): 19 per group, Gf (LPS): 27 per group). Therefore, insufficient power or training sessions do not explain why transfer effects could not be revealed in our study. In any case, including a sufficient number of subjects is important for any future study addressing training and transfer effects.

In accordance with no transfer effects, but against our hypothesis, a thorough examination of GM volume, MT and R2* in both a whole brain and a more sensitive region of interest analysis could not reveal differences between pretraining and posttraining MRI data. One of the most striking and earliest findings in favor of a link between practice and structural brain plasticity is provided by Draganski et al. (2004), who could show that a 3 months juggling training in younger adults led to GM changes within the mid‐temporal area and posterior intraparietal sulcus. Importantly, these behavioral and structural effects were transient since fluent juggling abilities and increases of GM volume returned to baseline 3 months after the training ended (see Boyke, Driemeyer, Gaser, Büchel, & May, 2008 for a replication in older adults). More recently, volume changes within the human motor cortex were described over the time course of 7 weeks (Wenger et al., 2017). Specifically, right‐handed human subjects practiced in nondominant, left‐hand writing and drawing, while up to 18 structural brain scans (MRI) were acquired. Importantly, increases in GM volume in the primary motor cortices were most pronounced after 4 weeks and they were no longer reliable after another 3 weeks despite still increasing task performance. Therefore, experience‐dependent structural brain changes appear to progress in a nonlinear fashion, which nicely fits to the “overproduction‐pruning” model, suggesting a fast increase of synapses only at the beginning of the intervention and a return to baseline over time (Lindenberger et al., 2017). With regard to our findings, more brain scans throughout the training period may have helped to identify possible nonlinear structural brain changes. Although the absence of transfer effects at the end of the training may argue against it, the clear improvement over time in the training task may be associated with nonlinear structural plasticity. In any case, time‐series sampling at the neural (and possibly behavioral) level may be considered in future studies.

Alternatively, the improvements of our 4‐week working memory training may only be associated with functional but not structural changes. For instance, based on BOLD fMRI, the study by Heinzel et al. (2016) found reduced hemodynamic activity in frontal brain regions associated with working memory processing, suggesting training‐related increases in processing efficiency. While structural brain changes may need more time to occur, the above‐mentioned study by Wenger et al. (2017) shows that, in principle, brain changes can be detected within 4 weeks. Future studies, therefore, may focus on both functional and anatomical brain changes.

Novel movie clips throughout the training did not further promote training gains or transfer effects. This assumption was based on the notion that novelty activates the dopaminergic mesolimbic system and thereby drives plasticity, including learning and memory (Lisman et al., 2011; Lisman & Grace, 2005). In humans, for instance, novel scene images improved subsequent memory (Fenker et al., 2008), and the exploration of a novel virtual reality (VR), compared to a familiar VR, enhanced recall (Schomaker et al., 2014). These effects are comparable with animal studies showing that long‐term memory is not only promoted through novelty exploration before but also after learning (Ballarini et al., 2009; Li et al., 2003; Moncada & Viola, 2007; Wang et al., 2010). At the neural level, novelty activates the SN/VTA, striatum, and the hippocampus (Bunzeck et al., 2014; Bunzeck & Düzel, 2006; Herweg, Sommer, & Bunzeck, 2018; Zaehle et al., 2013) providing further evidence for a link between dopaminergic neuromodulation and novelty.

Along the same lines, an impoverished environment (i.e., lack of social or physical stimuli) can lead to cognitive decline in animals and humans (see as a review Volkers & Scherder, 2011), further suggesting a positive effect of novelty. Importantly, older animals were more affected by impoverished environments than young animals (Bell, Livesey, & Meyer, 2009), but, interestingly, these negative effects seemed to be reversible when resettled to an enriched environment (Winocur, 1998). In humans, complex work environments may increase intellectual abilities and this effect was even higher in older than young workers (Schooler, Mulatu, & Oates, 1999). The importance of experiencing novelty at work (i.e., work‐task changes) has been further underlined by a positive correlation with processing speed, working memory, and GM volume in older adults (Oltmanns et al., 2017). Finally, a longitudinal study revealed that complex leisure time activities (e.g., reading books, visits of art institutions, or hobbies) increased intellectual functioning in older adults, while less complex activities led to reverse effects (Schooler & Mulatu, 2001). Together, these findings underline the importance of stimulating and novel environments in the context of age‐related plasticity.

Although our nature movies were rated as more positive compared to familiar ones there was no beneficial effect of novelty on performance in the training task or transfer effects. To further understand this issue, we already conducted an additional behavioral study (Biel & Bunzeck, 2019), in which young participants were exposed to novelty (same movies as in the present study) before, directly after, or 15 min after encoding of a word‐list. In line with our findings here, novel movies were rated as more positive, but they had no effect on subsequent recognition. We concluded that a passive exposure to novelty is not sufficient in order to promote plasticity and learning. Instead, a sense of agency, for instance through active choices during novelty processing, may be required (Murty, DuBrow, & Davachi, 2015). Therefore, a parsimonious explanation is that a stronger sense of agency, possibly associated with the engagement of memory related brain regions, is necessary in order to induce a positive effect of novelty on training gains and transfer effects.

After the training, task performance was significantly enhanced for most tests in both the novelty and familiarity group. However, such pre versus post differences were also significant in the passive control group after 4 weeks of no training, excluding the possibility of a specific training‐related transfer effect (Figure 5). Therefore, a more likely explanation relates to retesting, which has previously been described (Scharfen, Peters, & Holling, 2018). Specifically, repeated administration of a cognitive ability test can lead to an improvement of a third of a standard deviation, which may relate to practice rather than the measured ability itself (Lievens, Reeve, & Heggestad, 2007). Further, changes in confounding factors like anxiety and test familiarity at the second appointment can influence better test results (Reeve, Heggestad, & Lievens, 2009). Together, observing similar pre vs. post differences in our intervention groups and the passive control group can best be explained by retest effects. From a more general perspective, this highlights the importance of a passive control group in cognitive training studies in order to correctly assess training benefits.

The importance of interindividual differences in training studies has previously been highlighted (e.g., Buitenweg et al., 2012). Specifically, Jaeggi, Buschkuehl, Shah, and Jonides (2014) suggested that the effects of a working memory training may depend on factors including motivation, need for cognition, preexisting abilities and implicit theories about intelligence. Further, in another study with young participants, the success of a 6‐week juggling training (i.e., learning slopes) correlated with GM volumes within medial occipito‐parietal brain regions at baseline (Sampaio‐Baptista et al., 2014). Therefore, we investigated the relationship between training effects (gains and transfer, respectively) and cognitive abilities (MoCA), training effects, and personality traits (Big‐Five), as well as training effects and baseline structural integrity. None of these correlations, which were collapsed across the experimental groups and therefore included more than 50 subjects, revealed statistically significant effects. These analyses may not include all possible factors mentioned above, but they suggest that a relationship between individual differences and training gains may be more complex than previously thought.

Finally, a potential limitation of many training studies relates to the abstract nature of assessing cognitive functioning in older adults via standardized tests in a laboratory environment. Although standardized tests are indispensable, measuring everyday functioning in older adults and importantly, possible effects of cognitive training on those, can be challenging. In order to address this issue, we implemented a ticket vending machine, mimicking everyday functioning. However, as stated above, no improvements could be revealed. Therefore, future training studies should target a wider battery of everyday functioning in a nonlaboratory environment (i.e., questionnaires addressing daily life abilities).

Together, in healthy older adults a 4‐week two‐back working memory training improved working memory abilities. However, these training gains were restricted to the trained task and did not transfer to other cognitive domains. Novelty presentation throughout the training, supposed to be associated with dopaminergic neuromodulation, did not further promote training gains or transfer effects. At the neural level, pretraining versus posttraining comparisons did not reveal any structural brain changes in GM, myelin or iron levels. Therefore, our findings are in line with several recent studies, indicating that brain plasticity is specific to the trained ability and associated structural brain changes may be nonlinear.

## CONFLICT OF INTEREST

The authors declare no competing interests.

## AUTHOR CONTRIBUTIONS

D.B. acquired the data. D.B. and N.B. designed the study. T.V. and N.J. programmed the training task. D.B., T.S., and N.B. analyzed the data. D.B. and N.B. wrote the article. All authors reviewed the manuscript.

## Data Availability

The data that support the findings of this study are available on reasonable request from the corresponding authors (DB or NB). The data are not publicly available due to data security regulations by the local ethics committee.
